# Clinical and microbiological features of a cohort of patients with *Acinetobacter baumannii* bloodstream infections

**DOI:** 10.1007/s10096-024-04881-0

**Published:** 2024-07-18

**Authors:** Chongyang Wu, Yu Yuan, Sishi Tang, Chen Liu, Chao He

**Affiliations:** Department of Laboratory Medicine, West China Hospital, Sichuan University, Chengdu, Sichuan 610041 China

**Keywords:** *Acinetobacter baumannii*, Resistance, Bloodstream infection, Sequence type 2, Clustered regularly interspaced short palindromic repeats (CRISPR)

## Abstract

**Purpose:**

*Acinetobacter baumannii* is emerging as a pathogen that is a focus of global concern due to the frequent occurrence of the strains those are extensively resistant to antibiotics. This study was aimed to analyze the clinical and microbiological characteristics of a cohort of patients with *A. baumannii* bloodstream infections (BSIs) in western China.

**Methods:**

A retrospective study of the patients at West China Hospital of Sichuan University with *A. baumannii* BSIs between Jan, 2018 and May, 2023 was conducted. Antimicrobial susceptibility of *A. baumannii* isolates was tested by microdilution broth method. Whole-genome sequencing and genetic analysis were also performed for these isolates.

**Results:**

Among the 117 patients included, longer intensive care unit stay, higher mortality, and more frequent invasive procedures and use of more than 3 classes of antibiotics were observed among the carbapenem-resistant *A. baumannii* (CRAB)-infected group (*n* = 76), compared to the carbapenem-susceptible *A. baumannii* (CSAB)-infected group (*n* = 41, all *P* ≤ 0.001). Twenty-four sequence types (STs) were determined for the 117 isolates, and 98.7% (75/76) of CRAB were identified as ST2. Compared to non-ST2 isolates, ST2 isolates exhibited higher antibiotic resistance, and carried more resistance and virulence genes (*P* < 0.05). In addition, 80 (68.4%) isolates were CRISPR-positive, showed higher antibiotic susceptibility, and harbored less resistance and virulence genes, in comparison to CRISPR-negative ones (*P* < 0.05). Phylogenetic clustering based on coregenome SNPs indicated a sporadic occurrence of clonal transmission.

**Conclusion:**

Our findings demonstrate a high frequency of ST2 among *A. baumannii* causing BSIs, and high antibiotic susceptibility of non-ST2 and CRISPR-positive isolates. It is necessary to strengthen the surveillance of this pathogen.

**Supplementary Information:**

The online version contains supplementary material available at 10.1007/s10096-024-04881-0.

## Introduction

*Acinetobacter baumannii* has emerged as an important pathogen that causes infectious diseases such as pneumonia, bloodstream infections (BSIs), meningitis, and skin or soft tissue infections [1]. In recent years, there has been a sharp decline in the in vitro susceptibility to antibiotics among worldwide isolates of *A. baumannii* [2]. Critically, carbapenem-resistant *A. baumannii* (CRAB) is only susceptible to a small number of antimicrobial agents, which raises a worldwide health concern about the effective treatment of this pathogen [3, 4].

The molecular mechanisms of antimicrobial resistance and virulence of *A. baumannii* continue to be fully understood. The production of carbapenem hydrolases (e.g., OXA-23) is a key mechanism in the development of carbapenem resistance [5]. The *pmrA, pmrB* and *mcr* genes are related to colistin resistance [6]. Acquisition of the *tetX* gene and over-expression of resistance-nodulation-cell division efflux pumps have been observed in tigecycline-resistant strains [7, 8]. Virulence factors have also been identified, including outer membrane proteins [9], capsular polysaccharides [10], phospholipases [11] and acinetobactin-mediated iron acquisition system [12].

The occurrence of antimicrobial resistance and virulence genes or genetic elements, such as *bla*_OXA−23_ and *bla*_OXA−72_ [13], lipooligosaccharide genes in the outer core locus 1 (OCL1) [14], and genes involved in type VI secretory system (T6SS) [15], differed among *A. baumannii* strains causing BSIs. Also, various sequence types (STs) were identified among these isolates, and their relationship with the prognosis was variable. For example, the 30-day mortality was high in ST191, but rather low in ST451 [16]. ST191/195/208 strains prevailed in the patients with severe infections, demonstrated increased multidrug antimicrobial resistance, and caused excessive mortality, compared to the other stains [17]. Further research is needed to fully elucidate the clinical features of *A. baumannii* BSIs and to determine the antimicrobial susceptibility patterns and genotypes of the isolates, in order to guide optimal management of the patients. Therefore, the present study was aimed to identify the clinical and microbiological characteristics of a cohort of patients with *A. baumannii* BSIs.

## Materials and methods

### Patient inclusion and data collection

A retrospective study was conducted in the West China Hospital of Sichuan University, in Chengdu, China, according to the STROBE guidelines (Strengthening the Reporting of Observational Studies in Epidemiology) [18]. Medical data from patients suffering from *A. baumannii* BSIs between Jan 1st, 2018, and May 31st 2023 were retrieved from the hospital information system. *A. baumannii* BSI was defined as isolation of the bacterium from blood culture during the study period. Each case was ensured by the clinicians that the primary infection sites met the National Healthcare Safety Network (NHSN) definitions [19]. At last, 117 cases were included for further analysis. According to the in vitro susceptibility of *A. baumannii* isolates to carbapenem, the included cases were divided into a CRAB-infected group (*n* = 76) and a carbapenem-susceptible *A. baumannii* (CSAB)-infected group (*n* = 41). The flowchart for the patient inclusion and exclusion is summarized in Supplementary material Figure S1.

### Bacterial identification and whole-genome sequencing

*A. baumannii* strains isolated from the patients included were recovered on Luria-Bertani agar and incubated overnight at 37℃. The isolates were identified by a matrix-assisted laser desorption ionization-time of flight mass spectrometry (MALDI-TOF MS) Biotyper^®^ Sirius System (Bruker Daltonics GmbH, Bremen, Germany), and confirmed via whole genome-sequencing using an Illumina NovaSeq 6000 platform (Illumina, San Diego, CA, USA). *Escherichia coli* ATCC25922 and *A. baumannii* ATCC19606 were used as control strains.

### Antimicrobial susceptibility testing (AST) of the isolates

The in vitro susceptibility of the isolates to antibiotics was determined using a Vitek 2 compact system (bioMérieux, Lyon, France) according to the manufacturer’s recommendations. The results of minimum inhibitory concentration (MIC) were interpreted by the Clinical and Laboratory Standards Institute supplement M100-Ed33 [20]. *E. coli* ATCC25922 and *Pseudomonas aeruginosa* ATCC27853 acted as control strains.

### Genomic analysis of the isolates

Raw data from the whole-genome sequencing were subjected to quality control check using FastQC v0.12 (https://www.bioinformatics.babraham.ac.uk/projects/fastqc/), and the trimmed reads were assembled by SPAdes v3.15.5 [21]. Genome sequences were annotated by Prokka v1.1 [22]. Acquired resistance genes were identified by ResFinder v4.0 [23] using the CARD database (https://card.mcmaster.ca/). Virulence genes were identified by ABRicate v0.9.8 **(**https://github.com/tseemann/abricate) using the VFDB database (http://www.mgc.ac.cn/VFs/). The Clustered Regularly Interspaced Short Palindromic Repeats (CRISPR) arrays were searched by the CRISPRCasFinder v4.3.2 [24], CRISPRDetect v2.2 [25], and CRISPROne (https://omics.informatics.indiana.edu/CRISPRone/index.php). In this study, the strains were classified into CRISPR-positive or CRISPR-negative group according to the presence or absence of CRISPR array. The presence of *cas* genes was determined with NCBI RefSeq and TIGRFAM databases. And then, the type of CRISPR-Cas system was assigned [26].

### Phylogenetic analysis of the isolates

Multi-locus sequence typing (MLST) was performed on the PubMLST (https://pubmlst.org/organisms). A sequence type (ST) code for each isolate was generated based on the Pasteur scheme, according to the detection results of the allelic genes *cpn60, fusA, gltA, pyrG, recA, rplB*, and *rpoB*. A total of 1871 core genes was identified from the pan-genome analysis by Roary [27]. To determine phylogenetic relationships among the isolates, the genomic sequences were aligned to a complete reference genome *A. baumannii* ATCC 17978 (accession no. GCA_000015425.1), then the single nucleotide polymorphisms (SNPs) variant types (snp, ins, del, complex) were determined using Snippy v4.6.0 [28]. The recombination events were filtered using Gubbins v2.4.1 (https://github.com/nickjcroucher/gubbins) under the GTRGAMMA model (https://github.com/tseemann/snippy). SNP-sites [29] were used to extract SNPs from the recombination-free multi-FASTA alignment, resulting 176,740 core SNPs. A pairwise SNP distance matrix was generated by SNP-dists v0.8.2 (https://github.com/tseemann/snp-dists). The phylogenetic tree based on maximum-likelihood method was built with RAxML [30] and visualized using the iTOL online server (https://itol.embl.de/). Clonal transmission occurred when single nucleotide variations (SNVs) ≤ 10 between the isolates [31]. To identify the main genetic branches and trace the relationship of ST2 versus non-ST2 isolates, and CRISPR-positive versus CRISPR-negative ones, the phylogenetic trees were constructed, respectively.

### Statistical analysis

Continuous variables (e.g. ages, days) were expressed as the median with interquartile range (IQR), and categorical variables as frequency and percentage. The association between patients and bacterial characteristics was assessed with chi-squared tests for categorical variables and t-tests or Wilcoxon tests for continuous variables. Adjusted odds ratios (aOR) and 95% confidence intervals (CIs) were obtained from a multivariable logistic regression model. Data were analyzed using SPSS 26.0 software (SPSS Inc, USA) and a two-side *P* < 0.05 was considered statistically significant.

### Ethics declarations

Ethical permission was obtained by approval of the institutional review board of the West China Hospital of Sichuan University Clinical Trial center (No.2020 -954), in accordance with the International Guideline for Ethical Review of Epidemiological Studies and Declaration of Helsinki. The informed consent was waived because this study was a retrospective study with review of related data through the electronic medical records.

## Results

### Characteristics of *A. baumannii* BSIs

The demographics, clinical and microbiological features of the cases in the CRAB-infected (*n* = 76) and CSAB-infected (*n* = 41) groups are summarized in Table 1. A high 7-day mortality (43.6%) was observed among the 117 patients, and the mortality of the CRAB-infected group was much higher than that of the CSAB-infected group (57.9% versus 17.1%, *P*< 0.001). Stay in intensive care unit (ICU), invasive procedures, and the usage of more than 3 classes of antibiotics were more common in the CRAB-infected group, compared to the CSAB-infected group (all *P<* 0.05). Multivariate logistic regression analysis found that stay in ICU (aOR: 5.95; 95% CI 1.84-20.04; *P* = 0.003), nasogastric tube placement (aOR: 6.50; 95% CI 1.44-33.78; *P* = 0.018), mechanical ventilation (aOR: 13.50; 95% CI 5.34-49.5; *P* < 0.001), and usage of more than 3 classes of antibiotics (aOR: 3.40; 95% CI 1.19-10.10; *P* = 0.023) were the independent risk factors for the patients with CRAB BSIs.

**Table 1:** Demographics, clinical and microbiological features of *A. Baumannii* BSIs

Characteristics	CRAB-infected group (*n* = 76)	CSAB-infected group (*n* = 41)	*P* value
**Age (years, median (IQR))**	55.0 (48.0-71.0)	59.3 (33.0–69.0)	0.144
**Male, n (%)**	57 (75.0)	26 (63.4)	0.188
**Length of stay in hospital (days, median (IQR))**	18.4 (10.0–38.0)	17.9 (12.0-30.0)	0.800
**Stay in ICU, n (%)**	60 (78.9)	13 (31.7)	< 0.001
**Length of stay in hospital prior to positive culture (days, median (IQR))**	2.9 (2.0–10.0)	2.1 (2.0–11.0)	0.291
**Underlying conditions, n (%)**			
Hypertension	23 (30.3)	8 (19.5)	0.209
Diabetes	13 (17.1)	10 (24.4)	0.344
Cardiovascular disease	27 (35.5)	10 (24.4)	0.217
Chronic liver disease	37 (48.7)	15 (36.6)	0.209
Chronic kidney disease	39 (51.3)	19 (46.3)	0.608
Neurological disease	2 (2.6)	4 (9.8)	0.096
Solid cancer	4 (5.3)	6 (14.6)	0.084
Solid organ transplantation	4 (5.3)	1 (2.4)	0.471
**Invasive procedures, n (%)**			
Nasogastric tube placement	71 (93.4)	20 (48.8)	< 0.001
Urinary catheterization	72 (94.7)	25 (61.0)	< 0.001
Intravascular catheterization	68 (89.5)	26 (63.4)	0.001
Mechanical ventilation	72 (94.7)	19 (46.3)	< 0.001
Surgery	56 (73.7)	22 (53.7)	0.028
***A. baumannii *** **isolated from respiratory tract, n (%)**	33 (43.4)	11 (26.8)	0.077
**Usage of more than 3 classes of antibiotics, n (%)**	70 (92.1)	21 (51.2)	< 0.001
**Antibiotic regimens after positive blood culture, n (%)**			
Carbapenem	57 (75.0)	19 (46.3)	0.002
Cefoperazone/sulbactam	13 (17.1)	17 (41.5)	0.004
Tigecycline	27 (35.5)	6 (14.6)	0.017
Colistin	5 (6.6)	2 (4.9)	0.711
Tigecycline+Colistin	20 (26.3)	0	< 0.001
***A. baumannii *** **isolated from blood culture, n (%)**			
ST2 identified	75 (98.7)	1 (2.4)	< 0.001
Carrying more than 3 kinds of resistance genes	75 (98.7)	0	< 0.001
Carrying more than 3 kinds of virulence genes	76 (100)	41 (100)	1
CRISPR-positive isolates	42 (55.3)	38 (92.7)	<0.001
**7-day mortality, n (%)**	44 (57.9)	7 (17.1)	<0.001

*Abbreviations* CRAB, carbapenem-resistant *Acinetobacter baumannii*; CSAB, carbapenem-susceptible *Acinetobacter baumannii*; IQR, interquartile range; ICU, intensive care unit; ST, sequence type; CRISPR, Clustered Regularly Interspaced Short Palindromic Repeats. *Definitions* (1) *A. baumannii* BSI,the isolation of *A. baumannii* from blood culture of the cases ensured by the clinicians that the primary infection sites met the National Healthcare Safety Network (NHSN) definitions; (2) Length of stay in hospital, the number of days that patients spent in hospital from admission to discharge; (3) Stay in ICU, the admission to ICU prior to positive blood culture; (4) Invasive procedures, the administration of the nasogastric tube placement, urinary catheterization, intravascular catheterization, mechanical ventilation, or surgery one week prior to positive blood culture; (5) *A. baumannii *isolated from respiratory tract, the isolation of* A. baumannii* from respiratory tract specimens prior to positive blood culture (6) Usage of more than 3 classes of antibiotics, the administration of more than 3 classes of antibiotics prior to positive blood culture; (7) 7-day mortality, the ratio of the death during 7 days after positive blood culture

### Antimicrobial resistance phenotype and genomic features of CRISPR-positive and CRISPR-negative *A. baumannii* isolates

**Fig. 1:**
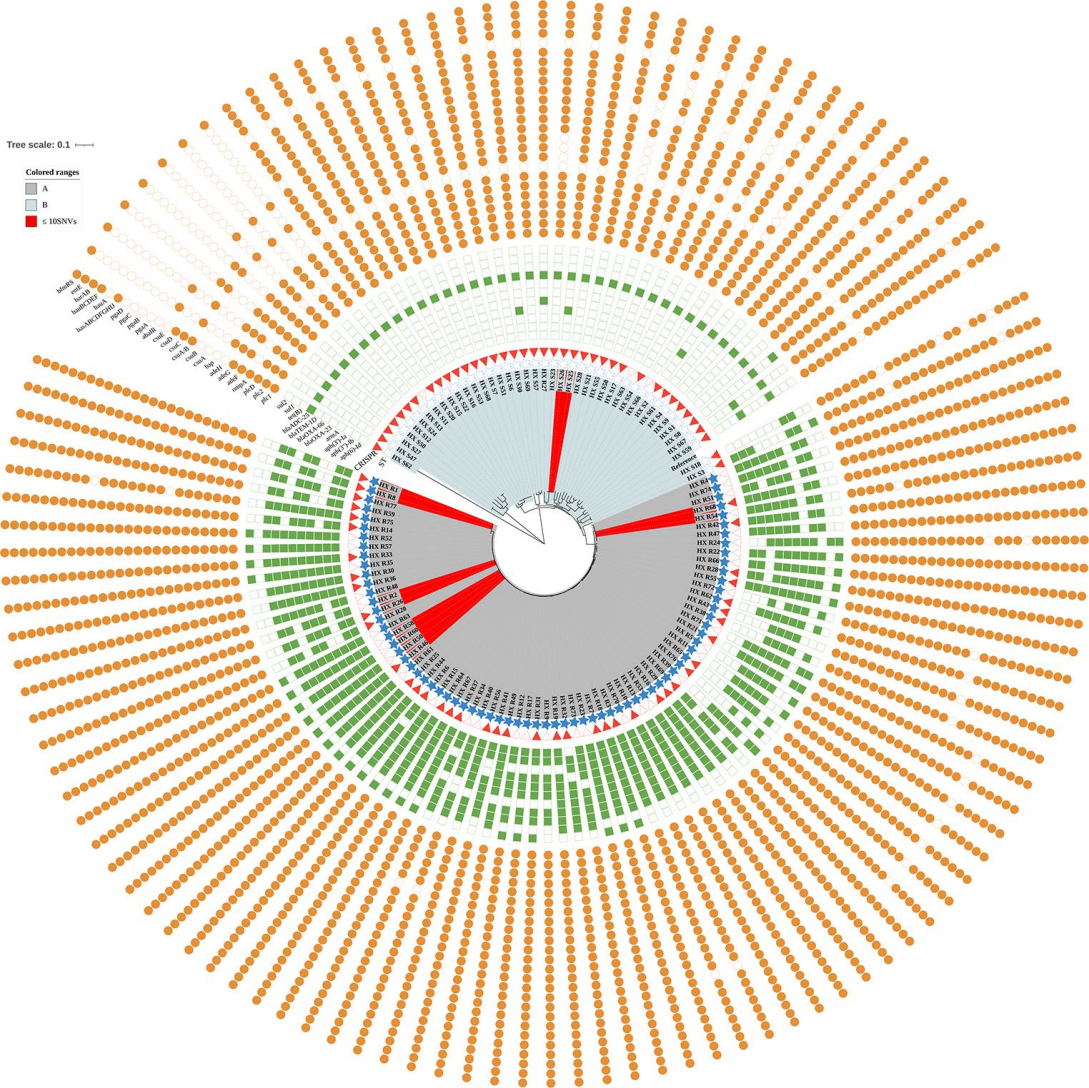
Phylogenetic analysis, heatmap of the resistance and virulence genes of *A. baumannii* isolates. The colored tracks adjacent to the tip of the phylogenetic tree show the different resistance and virulence genes, ST2 (blue stars) or non-ST2 (white stars) isolates, CRISPR-positive (red triangles) or CRISPR-negative (white triangles) isolates. The presence of each antibiotic resistance and virulence gene is shown with green square and orange circle respectively, while their absence with white one. Twelve isolates with closest genetic relationship are filled with red. *Abbreviations* CRISPR, Clustered Regularly Interspaced Short Palindromic Repeats; ST, sequence type

**Fig. 2:**
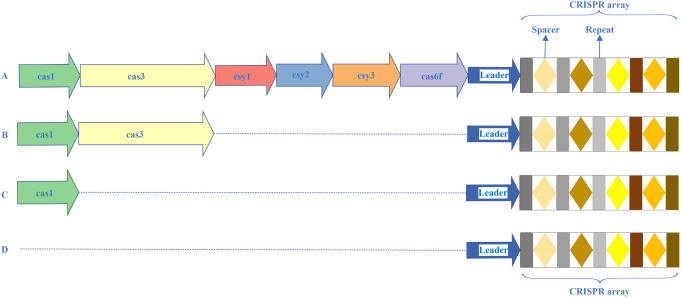
Genetic structures of Clustered Regularly Interspaced Short Palindromic Repeats (CRISPR) arrays identified in 80 *A. baumannii* isolates. (**A**) CRISPR-Cas type I-F1, *n* = 3; (**B**) CRISPR array + *cas1* + *cas3*, *n* = 6; (**C**) CRISPR array + *cas1*, *n* = 1; (**D**) CRISPR array, *n* = 70

**Fig. 3:**
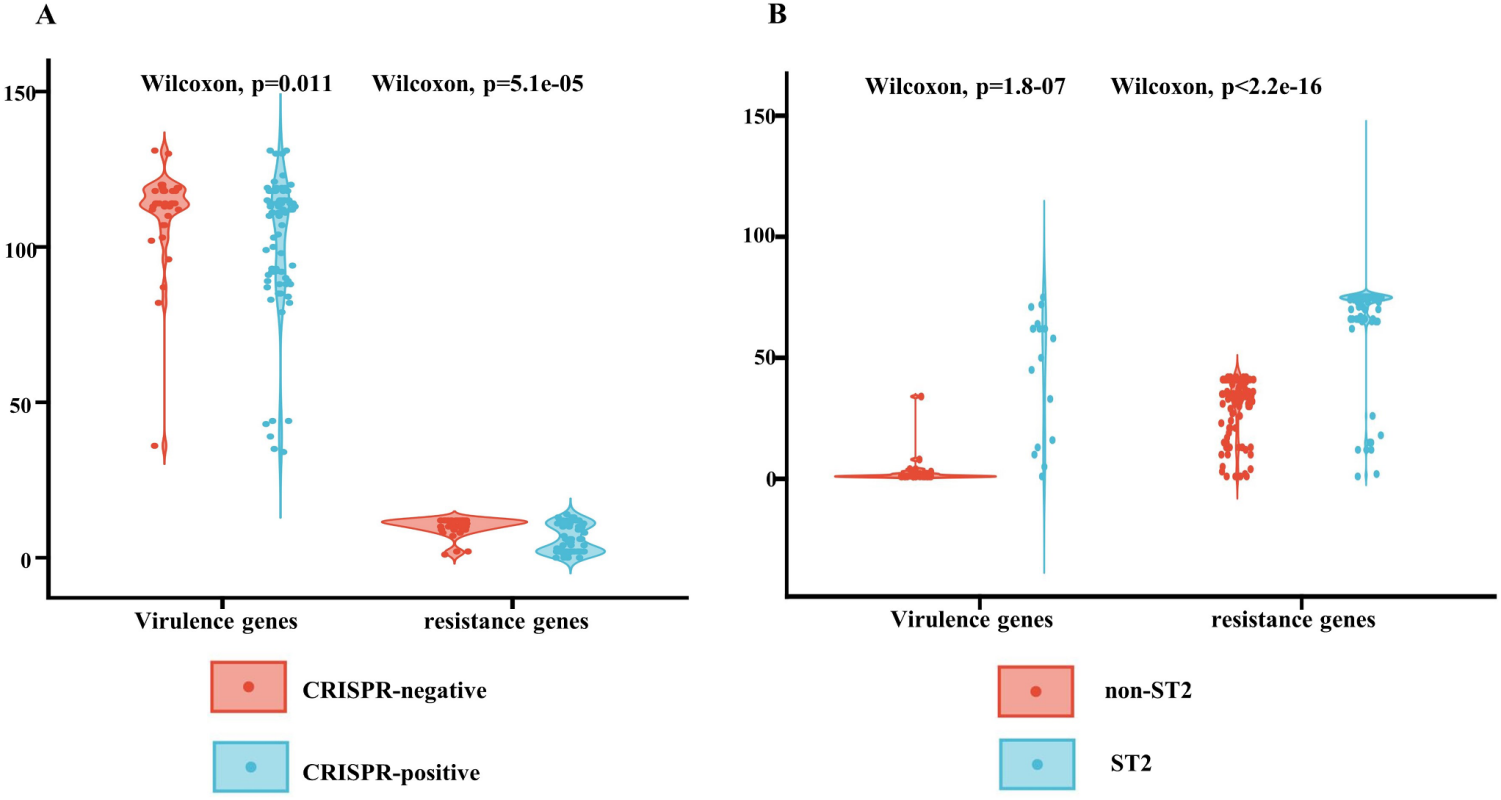
Comparative analysis of the resistance and virulence genes of *A. baumannii* isolates. (**A**) Comparison of the resistance and virulence genes between CRISPR-positive and CRISPR-negative *A. baumannii*. (**B**) Comparison of the resistance and virulence genes between ST2 and non-ST2 *A. baumannii*. *Abbreviations* CRISPR, Clustered Regularly Interspaced Short Palindromic Repeats; ST, sequence type

As demonstrated in Figs. 1 and 2, 68.4% (*n* = 80) of the 117 isolates carried CRISPR arrays. The CRISPR-negative isolates were more resistant to the antibiotics tested, compared to CRISPR-positive ones (Table 2, all *P* ≤ 0.001). Also, there were larger number of genes related to antimicrobial resistance and virulence among CRISPR-negative isolates, compared to CRISPR-positive ones **(**Fig. 3A; Tables 3 and 4).

**Table 2:** Antimicrobial resistance of CRISPR-positive/negative and ST2/non-ST2 *A. baumannii*

Antibiotics	Resistance (n, %)		*P* value	Resistance (n, %)		*P* value
	CRISPR-positive (*n* = 80)	CRISPR-negative (*n* = 37)		ST2 (*n* = 75)	non-ST2 (*n* = 42)	
β-lactams						
Ticacillin/clavulanic acid	43 (53.8)	36 (97.3)	< 0.001	75 (100)	4 (9.5)	< 0.001
Piperacillin/tazobactam	43 (53.8)	36 (97.3)	< 0.001	75 (100)	4 (9.5)	< 0.001
Ampicillin/sulbactam	43 (53.8)	36 (97.3)	< 0.001	75 (100)	4 (9.5)	< 0.001
Cefotaxime	43 (53.8)	36 (97.3)	< 0.001	75 (100)	4 (9.5)	< 0.001
Ceftriaxone	43 (53.8)	36 (97.3)	< 0.001	75 (100)	4 (9.5)	< 0.001
Ceftazidime	43 (53.8)	36 (97.3)	< 0.001	75 (100)	4 (9.5)	< 0.001
Cefepime	43 (53.8)	36 (97.3)	< 0.001	75 (100)	4 (9.5)	< 0.001
Imipenem	42 (52.5)	34 (91.9)	< 0.001	75 (100)	1 (2.4)	< 0.001
Meropenem	42 (52.5)	34 (91.9)	< 0.001	75 (100)	1 (2.4)	< 0.001
**Aminoglycosides**						
Amikacin	32 (40.0)	32 (86.5)	< 0.001	63 (84.0)	1 (2.4)	< 0.001
Gentamicin	32 (40.0)	34 (91.9)	< 0.001	64 (85.3)	2 (4.8)	< 0.001
Tobramycin	32 (40.0)	32 (86.5)	< 0.001	63 (84.0)	1 (2.4)	< 0.001
**Fluoroquinolones**						
Levofloxacin	40 (50.0)	32 (86.5)	< 0.001	68 (90.7)	4 (9.5)	< 0.001
Ciprofloxacin	43 (53.8)	33 (89.2)	< 0.001	72 (96.0)	4 (9.5)	< 0.001
**Tetracyclines**						
Tetracycline	38 (47.5)	34 (91.9)	< 0.001	68 (90.7)	4 (9.5)	< 0.001
**Folate pathway antagonists**						
Sulfamethoxazole	27 (33.8)	28 (75.7)	< 0.001	50 (66.7)	5 (11.9)	< 0.001

*Abbreviations* CRISPR, Clustered Regularly Interspaced Short Palindromic Repeats; ST, sequence type

**Table 3:** Antimicrobial resistance genes in CRISPR-positive/negative and ST2/non-ST2 *A. baumannii*

Antimicrobial resistance genes	Genes detected (n, %)		*P* value	Genes detected (n, %)		*P* value
	CRISPR-positive (*n* = 80)	CRISPR-negative (*n* = 37)		ST2 (*n* = 75)	non-ST2 (*n* = 42)	
**β-lactams**						
* bla*_*OXA−66*_	41 (51.3)	32 (86.5)	< 0.001	71 (94.7)	2 (4.8)	< 0.001
* bla*_*OXA−23*_	41 (51.3)	32 (86.5)	< 0.001	72 (96.0)	1 (2.4)	< 0.001
* bla*_*ADC−25*_	80 (100)	37 (100)	1	75 (100)	42 (100)	1
* bla*_*TEM−1D*_	27 (33.8)	24 (64.9)	0.002	50 (66.7)	1 (2.4)	< 0.001
**Aminoglycosides**						
* armA*	34 (42.5)	37 (100)	< 0.001	64 (85.3)	7 (16.7)	< 0.001
* aph(3’’)-Ib*	35 (43.8)	28 (75.7)	0.001	62 (82.7)	1 (2.4)	< 0.001
* aph(6)-Id*	35 (43.8)	28 (75.7)	0.001	62 (82.7)	1 (2.4)	< 0.001
* aph(3’)-Ia*	24 (30.0)	22 (59.5)	0.002	45 (60.0)	1 (2.4)	< 0.001
**Tetracycline**						
* tet(B)*	37 (46.3)	31 (83.8)	< 0.001	66 (88.0)	2 (4.8)	< 0.001
**Sulfonamides**						
* sul1*	9 (11.3)	7 (18.9)	0.262	16 (21.3)	0 (0)	0.002
* sul2*	16 (20.0)	19 (51.4)	0.001	33 (44.0)	2 (4.8)	< 0.001

*Abbreviations* CRISPR, Clustered Regularly Interspaced Short Palindromic Repeats; ST, sequence type

**Table 4:** Virulence-related genes in CRISPR-positive/negative and ST2/non-ST2 *A. baumannii*

Virulence factors	Related genes	Genes detected (n, %)		*P* value	Genes detected (n, %)		*P* value
		CRISPR-positive (*n* = 80)	CRISPR-negative (*n* = 37)		ST2 (*n* = 75)	non-ST2 (*n* = 42)	
**Exotoxin**							
Phospholipase	*plc1*	80 (100)	37 (100)	1	75 (100)	42 (100)	1
	*plc2*	80 (100)	37 (100)	1	75 (100)	42 (100)	1
	*plcD*	80 (100)	37 (100)	1	75 (100)	42 (100)	1
**Immune modulation factors**							
Outer membrane protein A	*ompA*	54 (67.5)	37 (100)	< 0.001	75 (100)	36 (85.7)	0.002
**Biofilm forming factors**							
AdeFGH efflux pump	*adeF*	80 (100)	37 (100)	1	75 (100)	42 (100)	1
	*adeG*	80 (100)	36 (97.3)	0.316	74 (98.7)	42 (100)	1
	*adeH*	77 (96.3)	37 (100)	0.551	73 (97.3)	41 (97.6)	1
Biofilm-associated protein	*bap*	43 (53.8)	34 (91.9)	< 0.001	73 (97.3)	4 (9.5)	< 0.001
Csu pili	*csuA*	71 (88.8)	36 (97.3)	0.167	75 (100)	32 (76.2)	< 0.001
	*csuB*	71 (88.8)	36 (97.3)	0.167	75 (100)	32 (76.2)	< 0.001
	*csuA/B*	77 (96.3)	37 (100)	0.551	75 (100)	39 (92.9)	0.044
	*csuC*	77 (96.3)	34 (91.9)	0.379	75 (100)	36 (85.7)	0.002
	*csuD*	77 (96.3)	32 (86.5)	0.107	75 (100)	34 (81.0)	< 0.001
	*csuE*	77 (96.3)	37 (100)	0.551	75 (100)	39 (92.9)	0.044
Quorom sensing system	*abaI*	61 (76.3)	30 (81.1)	0.559	70 (93.3)	21 (50.0)	< 0.001
	*abaR*	61 (76.3)	30 (81.1)	0.559	70 (93.3)	21 (50.0)	< 0.001
β-(1–>6)-Poly-N-acetyl-D-glucosamine (PNAG)	*pgaA*	78 (97.5)	37 (100)	1	74 (98.7)	31 (73.8)	< 0.001
	*pgaB*	73 (91.3)	33 (89.2)	0.740	73 (97.3)	33 (78.6)	0.002
	*pgaC*	78 (97.5)	37 (100)	1	75 (100)	30 (71.4)	< 0.001
	*pgaD*	73 (91.3)	37 (100)	0.096	67 (89.3)	33 (78.6)	0.113
**Nutritional/Metabolic factors**							
Acinetobactin	*basABCDFGHIJ*	74 (92.5)	36 (97.3)	0.429	75 (100)	35 (83.3)	0.001
	*bauA*	42 (52.5)	34 (91.9)	< 0.001	74 (98.7)	2 (4.8)	< 0.001
	*bauBCDEF*	74 (92.5)	36 (97.3)	0.429	75 (100)	35 (83.3)	0.001
	*barAB*	74 (92.5)	36 (97.3)	0.429	75 (100)	35 (83.3)	0.001
	*entE*	74 (92.5)	36 (97.3)	0.429	75 (100)	35 (83.3)	0.001
**Regulation factors**							
Biofilm-controlling response regulator	*bfmRS*	80 (100)	37 (100)	1	75 (100)	42 (100)	1

*Abbreviations* CRISPR, Clustered Regularly Interspaced Short Palindromic Repeats; ST, sequence type

### Antimicrobial resistance phenotype and genomic features of ST2 and non-ST2 *A. baumannii* isolates

In total, 24 STs, namely ST2, ST25, ST40, ST46, ST93, ST106, ST203, ST216, ST217, ST331, ST374, ST410, ST452, ST516, ST584, ST768, ST1153, ST1264, ST1336, ST1399, ST1512, ST1641, ST2034, ST2114, were determined via the Pasteur scheme. ST2 isolates accounted for 64.1% (75/117) of all the isolates and 98.7% (75/76) of CRAB isolates. The in vitro susceptibility of ST2 isolates to antibiotics was lower than that of non-ST2 isolates (Table 2, *P<* 0.001). ST2 isolates carried more resistance and virulence genes, compared to non-ST2 ones (Fig. 3B; Tables 3 and 4).

### Phylogenetic analysis of *A. baumannii* isolates

**Fig. 4:**
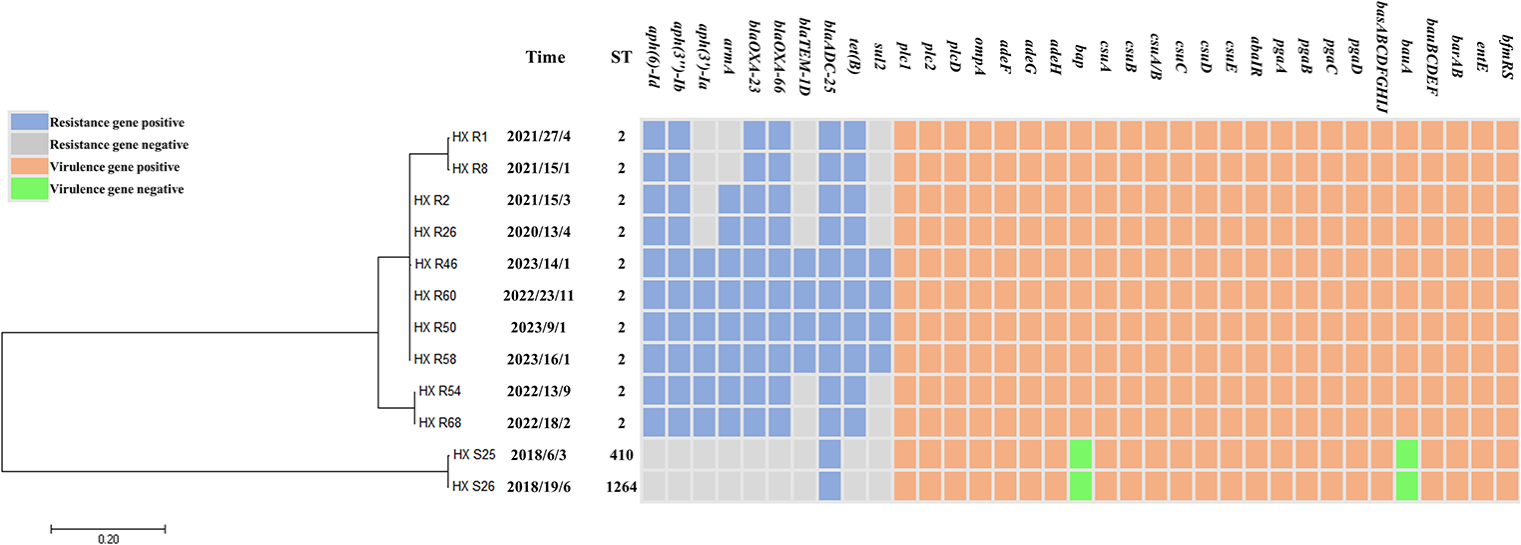
Phylogenetic analysis of 12 *A. baumannii* isolates with ≤ 10 single nucleotide variations (SNVs). Isolation time is indicated as yyyy/dd/mm. The colored tracks adjacent to the tip of the phylogenetic tree show the different resistance and virulence genes, and sequence types (STs)

The core-genome SNPs analysis of 117 *A. baumannii* isolates revealed an extensive genetic diversity, identifying 2 to 176,740 SNPs, 1,871 core genes that are common across all the strains, alongside a vast number of accessory (139,665) and unique (1,996) genes. Two clades, labeled as A and B in Fig. 1, were identified. The clade A included 75 ST2 (matrix distance: 2 ~ 720 SNPs), and the clade B had 42 non-ST2 ones (matrix distance: 3400 ~ 5474 SNPs). The closest genetic relationship was found among 10 ST2 CRAB and 2 non-ST2 CSAB isolates (Figs. 1 and 4), indicating clonal transmissions might occur.

Among the 75 ST2 isolates, a broad spectrum of genetic variation was characterized, including 3,355,802 variants (variant-SNPs 2,769,376, variant-insertions 30,120, variant-deletions 26,192, and variant-complex 530,114), which highlighted the prominence of ST2. The main genetic branches of ST2 versus non-ST2 isolates, and CRISPR-positive versus CRISPR-negative ones were shown in Figure S2-S5, respectively.

## Discussion

BSIs caused by multidrug-resistant bacteria are usually associated with the poor prognosis of the patients. In present study, a high 7-day mortality (43.6%) was observed among the patients with *A. baumannii* BSIs. Moreover, the mortality of CRAB-infected group was much higher than that of the CSAB-infected group (57.9% versus 17.1%). The high mortality (69.4%, 75/108) of CRAB BSIs was also observed previously [32]. All of our cases in the CRAB-infected group received invasive procedures, and 92.1% of these cases were administered with more than 3 classes of antimicrobial agents. Interestingly, we observed that *A. baumannii* was also isolated from respiratory tract in 43.4% (33/76) of cases in the CRAB-infected group and 26.8% (11/41) of cases in the CSAB-infected group. These data may be due to the colonization of *A. baumannii* in the respiratory tract as a crucial step that precedes the development of BSIs [33].

Recently, the increasing resistance of *A. baumannii* to antimicrobial agents has emerged as a global health concern. According to the China Antimicrobial Surveillance Network report (http://www.chinets.com), the resistance of *A. baumannii* to imipenem increased from 32.9% in 2005 to 71.2% in 2022. In our study, all of the CRAB isolates were resistant to multiple classes of antibiotics. The infections in other sites (e.g. respiratory tract) and variable comorbidities (as described in Table 1) might contribute to the high mortality among our CRAB-infected group, despite 68.4% of them were administrated with last-line antibiotics such as tigecycline and colistin. Similarly, the previous studies found that although tigecycline and colistin had not significantly reduced the death of patients with CRAB associated infections for prominent toxicity (both nephrotoxicity and neurotoxicity) and low plasma concentrations of the colistin contributing to failed treatments [34, 35]. Therefore, the prevention of CRAB BSIs may be critically important under such circumstances.

Furthermore, we identified the resistance phenotypes and associated genes of the isolates in this study. We found that 96.1% (73/76) of the CRAB isolates carried *bla*_OXA−23_, a gene that encodes a class D carbapenemase and contributes to a higher level of carbapenem resistance in *A. baumannii* [36]. The 3 isolates of *bla*_OXA−23_-negative CRAB carried *bla*_OXA−66,_ which is another kind of carbapenemase gene. The virulence factors identified in *A. baumannii* were mainly involved in immune modulation, biofilm formation, nutrition, metabolism, and regulation [37]. The *ompA* gene, encoding outer membrane protein A (OmpA) [38], was detected in all the CRAB isolates. The gene *bap*, contributing to biofilm production, cell adhesion, and invasion [39, 40], was identified in 97.4% (74/76) of CRAB isolates. The quorum sensing system *abaI/abaR*, as a signal transduction factor and acyl-homoserine lactone (AHL) synthase receptor [41], was detected in 93.4% (71/76) of CRAB isolates.

The CRISPR-Cas system is a form of bacterial immune protection against the invasion of mobile genetic elements [42]. CRISPR-positive isolates of *Klebsiella pneumoniae* have been shown to be more susceptible to antibiotics compared to CRISPR-negative ones [43]. In present study, CRISPR-positive * A. baumannii* isolates showed higher in vitro susceptiblity to antibiotics, and carried fewer resistance and virulence genes, compared to CRISPR-negative ones. This finding suggests that the CRISPR array may be a barrier to antimicrobial resistance in CSAB isolates, which might provide a new insight into the prevention and control of infections caused by this pathogen.

In our study, ST2 was identified in 64.1% of 117 *A. baumannii* isolates and 98.7% of 76 CRAB isolates. Other studies about *A. baumannii* BSIs reported that 52.0% of the isolates were identified as ST2 [44], and all the multidrug-resistant isolates as ST2 [32]. The dissemination of *A. baumannii* ST2 has attracted significant attention due to high resistance of the isolates and high mortality of the patients [44]. ST2 isolates showed multidrug resistance and harbored important virulence factors [45, 46]. Similarily, in our study, ST2 isolates carried more resistance and virulence genes, compared to non-ST2 ones. Furthermore, 12 isolates (with ≤ 10 SNVs) were found to be closely related genetically, which indicated that clonal transmission might occur.

There were some limitations in our study. It was a retrospective study conducted in a single center, and the sample size was small. Further research is needed to enlarge the sample size to find more genetic relationship among the *A. baumannii* isolates causing BSIs.

In conclusion, stay in ICU, nasogastric tube placement, mechanical ventilation, and usage of more than 3 classes of antibiotics were found to be the risk factors for CRAB BSIs. ST2 isolates exhibited higher antibiotic resistance, and carried more resistance and virulence genes, in comparison to non-ST2 ones. CRISPR-negative isolates were more resistant to antibiotics, and harbored more resistance and virulence genes, compared to CRISPR-positive ones. Phylogenetic clustering based on core-genome SNPs indicated a sporadic occurrence of clonal transmission. It is necessary to strengthen the surveillance of this pathogen.

### Electronic supplementary material

Below is the link to the electronic supplementary material.

Supplementary Material 1

## Data Availability

The data from this whole genome shotgun project have been deposited at the NCBI databases (Bio Project ID: PRJNA1014798 and PRJNA951345).
