# Supplementary material for: Clinical and microbiological features of a cohort of patients with Acinetobacter baumannii bloodstream infections
Source: Eur J Clin Microbiol Infect Dis. 2024 Jul 18;43(9):1721–30. doi: 10.1007/s10096-024-04881-0 (PMC11349859; doi:10.1007/s10096-024-04881-0)

**Figure S1 Flowchart of patient inclusion and exclusion in this study.**

**Figure S2 Phylogenetic analysis of ST2 *A. baumannii* isolates.**

**Figure S3 Phylogenetic analysis of non-ST2 *A. baumannii* isolates.**

**Figure S4 Phylogenetic analysis of CRISPR-positive *A. baumannii* isolates.**

**Figure S5 Phylogenetic analysis of CRISPR-negative *A. baumannii* isolates.**

**
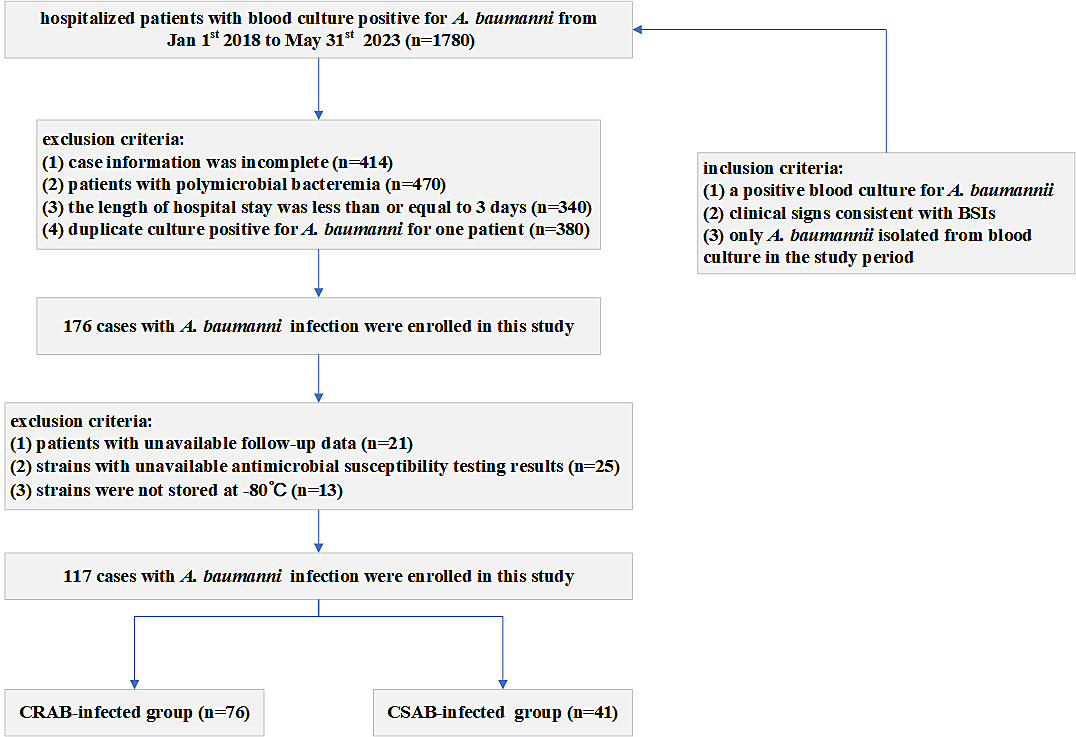
**


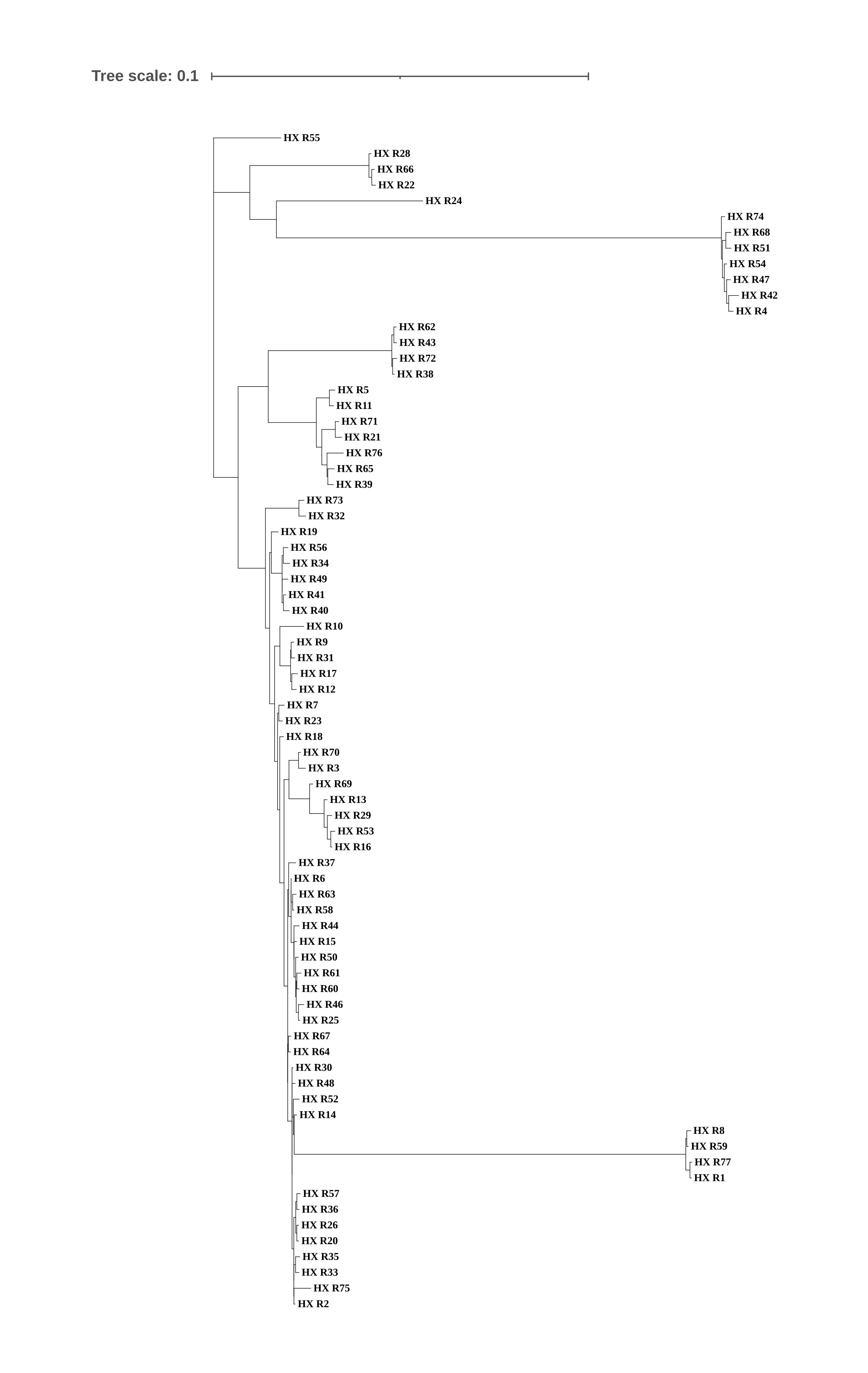

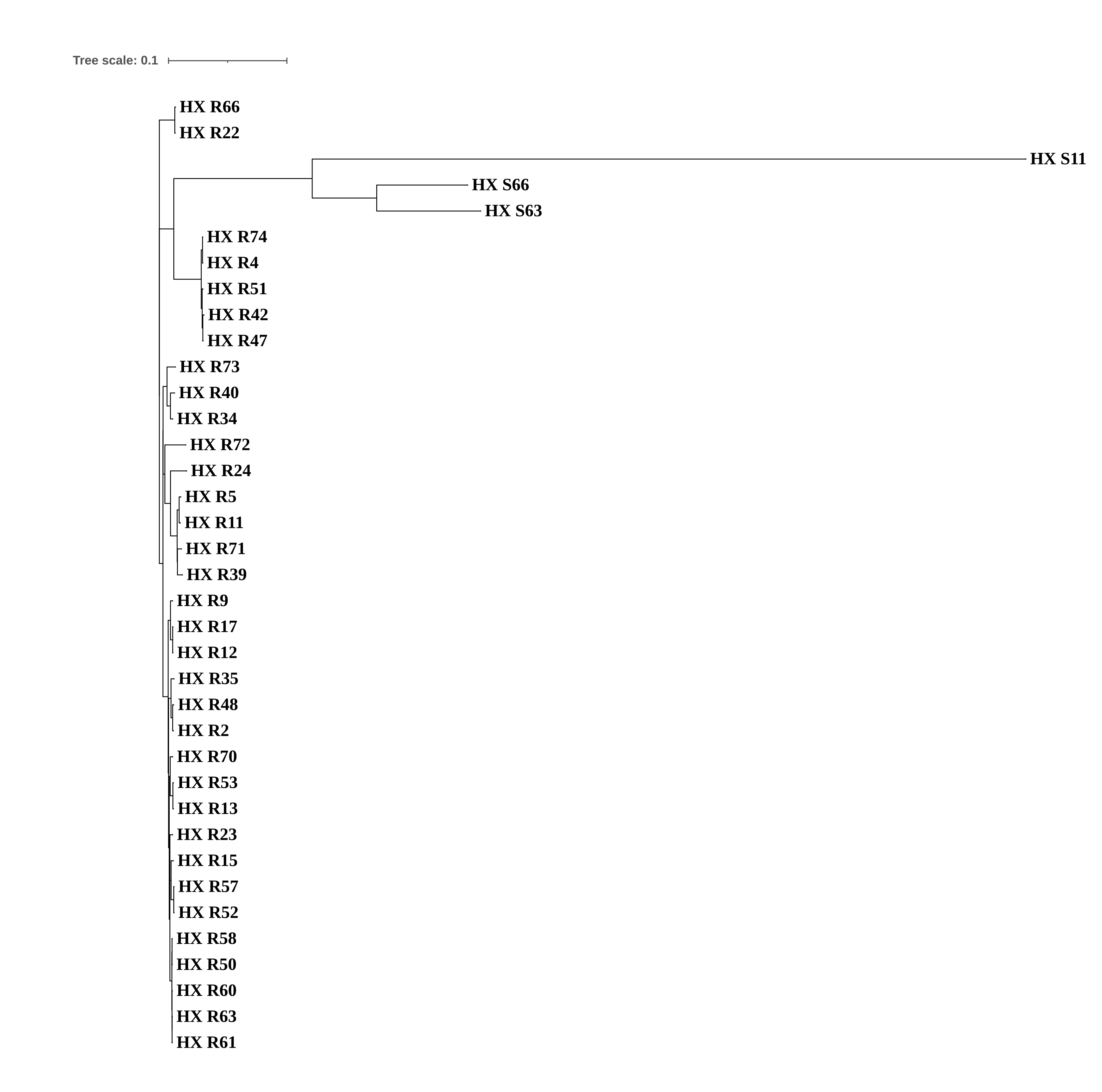

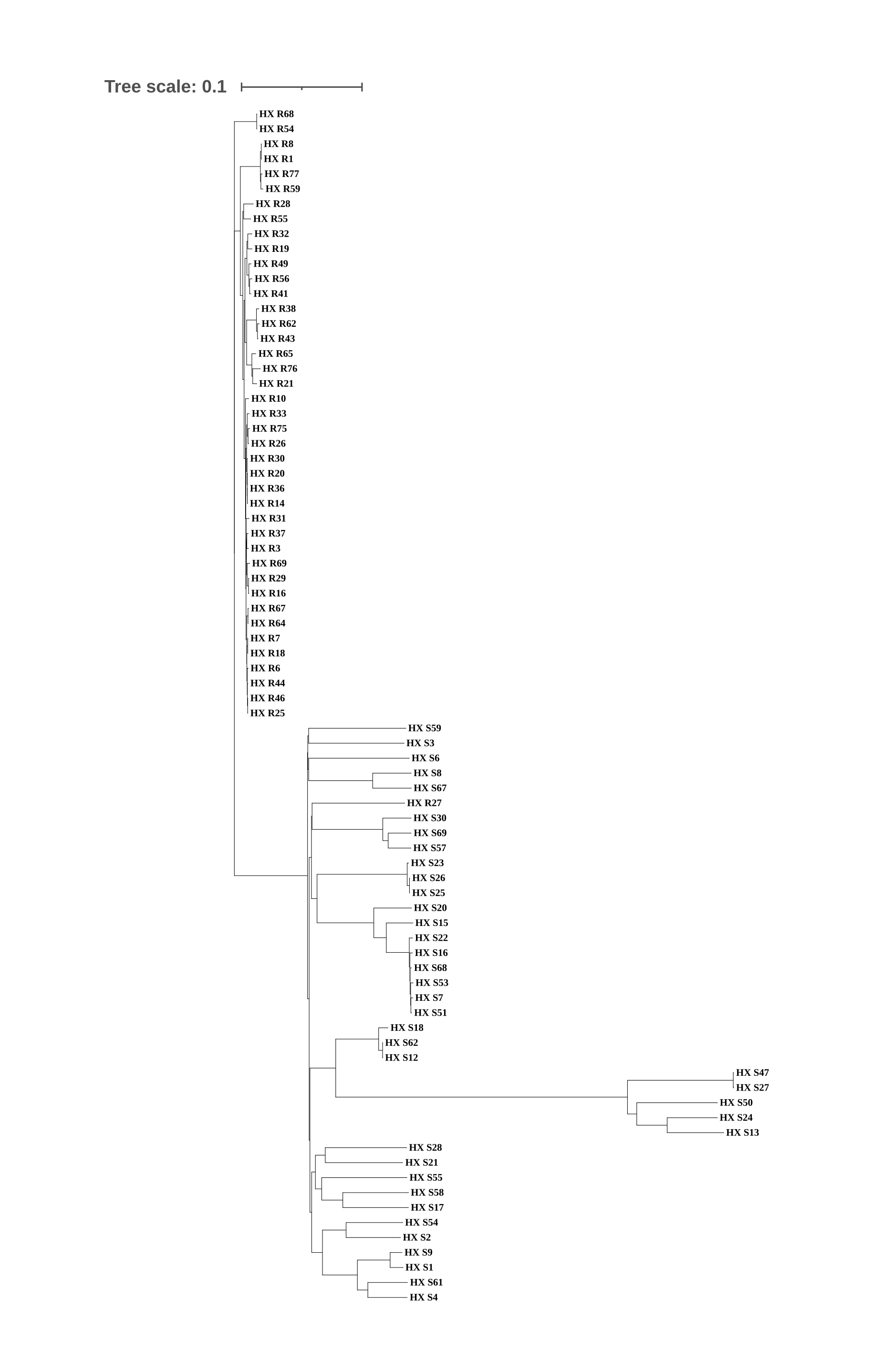

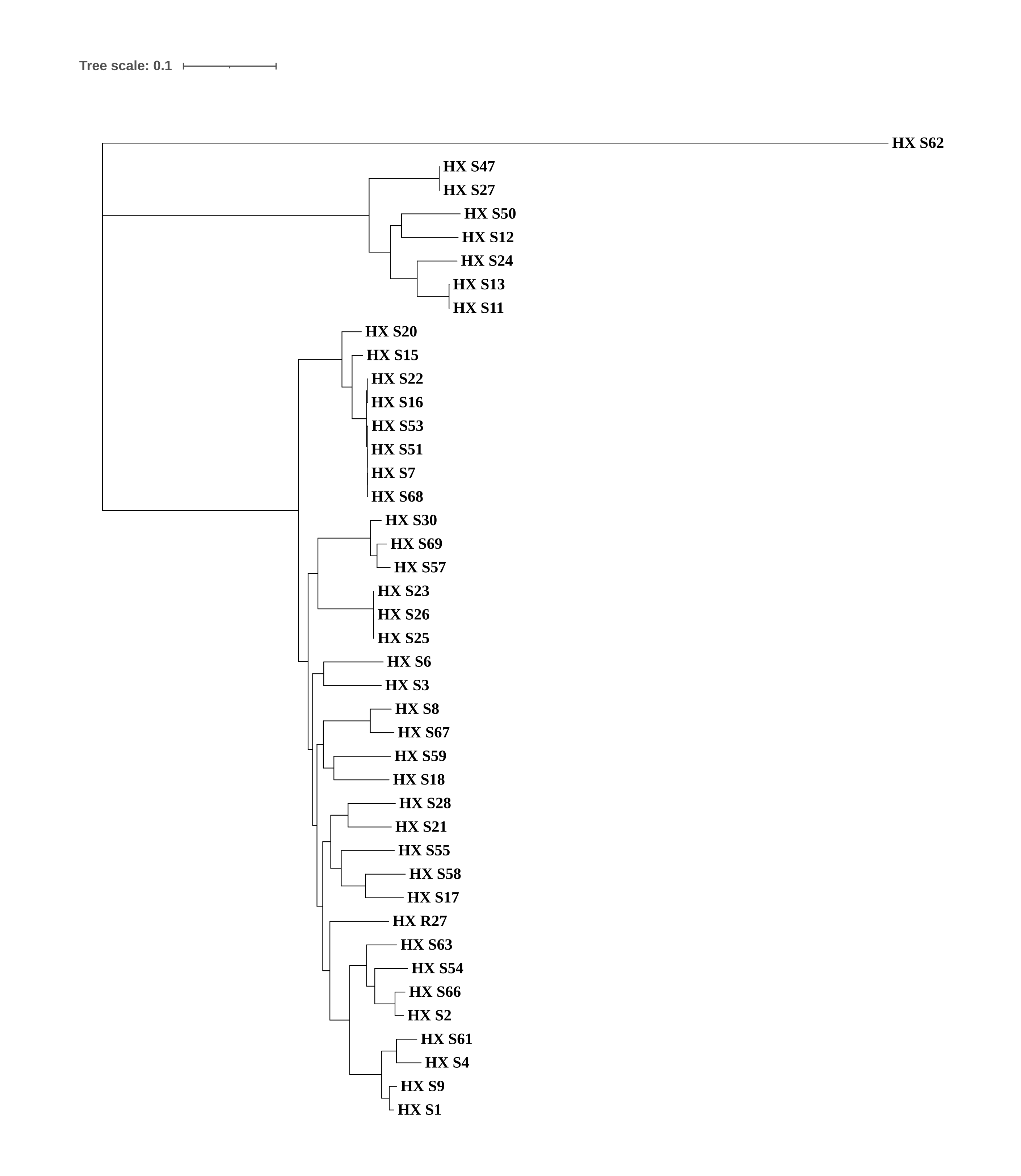

Supplement: Supplementary file 1 — Supplementary Material 1 [file 10096_2024_4881_MOESM1_ESM.docx]
